# Acknowledgement to Reviewers of *Extracellular Vesicles and Circulating Nucleic Acids* in 2022

**DOI:** 10.20517/evcna.2023.02

**Published:** 2023-01-28

**Authors:** EVCNA Editorial Office

**Affiliations:** Suite 1504, Tower A, Xi’an National Digital Publishing Base, No. 996 Tiangu 7th Road, Gaoxin District, Xi’an 710077, Shaanxi, China.

The editors of EVCNA would like to extend our gratitude and recognition to the following reviewers for their precious time and efforts in appraising the received manuscripts, regardless of whether the papers they reviewed were finally published in 2022 [Table 1].

**Table 1:** The reviewers in 2022

Badilescu Simona	Holliday Shannon	Sil Susmita
Balbi Carolina	Huang Zhaohui	Sistermans Erik A.
Bart Geneviève	Jiang Cheng	Soekmadji Carolina
Bhattacharyya Suvendra	Jiang Peiyong	Soper Steven A.
Bussolati Benedetta	Kim Dongin	Sundar Isaac Kirubakaran
Chandrananda Dineika	Kirubakaran Isaac	Thakur Abhimanyu
Cheng Yong	Li Guoping	Thakur Basant Kumar
Choi Hojun	Li Yong	Toh Wei Seong
Compton Carolyn	Liu Guozhen	Ullah Mujib
Cornel Martina	Lorenowicz Magdalena	Uysal-Onganer Pinar
Cui Xiaolin	Lu Yao	Verderio Claudia
D'AgostinoVito G.	Luo Yang	Vojtech Lucia
De la Cuesta Fernando	Matei Irina R.	Wang Xianwen
Deep Gagan	Meckes David	Wang Zhongyan
Dutta Suman	Mouliere Florent	White Ian M.
Eliceiri Brian P.	Nam Gi-Hoon	Xia Weiliang
Foot Natalie	Nolan John P.	Yasui Takao
Fortunato Orazio	Ochiya Takahiro	Yoshioka Yusuke
Garzia Livia	Pacini Luca	You Yang
Ghatak Subhadip	Puhka Maija	Zhang Da
Glebov Konstantin	Pulliam Lynn	Zhang Huang-ge
Graner Michael	Ragni Enrico	Zhou Feifan
Hakami Ramin M.	Rai Alin	Zhou Qing
Hau Peter	Seras-Franzoso Joaquin	
Henneman Peter	Sharma Pranav	

We greatly appreciate the contribution of expert reviewers, which is crucial to the journal’s editorial decision-making process. As a token of gratitude to our reviewers, in 2023, we will create an account for each of them in the submission system, and they can access details of their past reviews, see the comments of other reviewers, and download a letter of acknowledgement for their records. In addition, the Editorial Office will select two “Best Reviewers” in 2023 and each awardee will be awarded USD 350. Thanks again for their generous support in the past year.

## DECLARATIONS

### Copyright

©The Author(s) 2023.

